# Intussusception in Infants—Laparotomy and Exsection

**Published:** 1889-02

**Authors:** Albert B. Strong

**Affiliations:** Attending Surgeon to the Cook County Hospital, Chicago; 312 West Indiana Street


### CHICAGO MEDICAL

### JOURNAL AND EXAMINER.

VOLUME LVIII. FEBRUARY, 1889. NUMBER 2.

### TWO CASES OF INTUSSUSCEPTION IN INFANTS: LAPAROTOMY IN BOTH; INTESTINAL EXSECTION IN ONE.

BY ALBERT B. STRONG, M.D.,

ATTENDING SURGEON TO THE COOK COUNTY HOSPITAL, CHICAGO.

I was called at a late hour on the night of May 10, 1888, in consultation with Dr. E. E. Holroyd, to a case of supposed intestinal obstruction.

The history (as given by Dr. Holroyd) was as follows: A. C. W., a large, well-developed nursing infant 5½ months of age, who was first seen on the forenoon of May 8th. He had been ill several hours; vomiting and having stools at intervals of three or four hours. The first one was blood and the subsequent ones bloody, sero-mucus, and quite profuse. These had the peculiar odor of severe intestinal catarrh. There was a previous history of constipation; temperature 102°; pulse 130; respiration accelerated, but of natural rhythm. Abdomen normal, except a slight tenderness in the right inguinal region.

May 9th, at 9 A. M., temperature normal; pulse 100, respiration quite natural; vomited less frequently and takes the breast which the day before he refused. Two passages from the bowels since first visit, of similar appearance, but less profuse. At 9 P. M., his condition was unchanged.

I was called in at 8 A. M. the following morning as fæcal vomiting had occurred. I found the abdomen somewhat tense; detected a lump with tenderness in the right inguinal region. The father being out of town, I explained to the mother the probable condition of invagination, and administered two large enemata, but without benefit. An operation was then suggested, but I could not get the mother's consent. Temperature normal; pulse 140; respiration 40.

At 3 P. M. temperature 99°; pulse 150; respiration 40. Fæcal vomiting continued. No more stools.

At 8 P. M., temperature was 100°; pulse 150; respiration 40; vomiting; no stools. At midnight the father returned and consented to an operation.

An hour later when I saw the child, his condition was as follows: The general appearance indicated pain and approaching collapse. The face was slightly flushed; pupils widely dilated; eyelids twitching. The movements of the body were restless, the head being turned to and fro. The expression was peculiarly pinched in appearance. The abdomen was very much distended, tympanitic, and tender on pressure on the right side, where a small circumscribed tumor could be felt over the ileo-cæcal region.

Under a few whiffs of chloroform the abdomen was opened in the median line, when a small quantity of serum escaped.

There was no exudation of lymph, though the intestines and mesentery were markedly congested. The small intestine was distended to its greatest limit with gas and fluid contents. An unusually long mesentery was attached to the cæcum, so that it was easily lifted out of the abdominal cavity. The last fourteen inches of the ileum had passed through the ileo-cæcal valve, completely distending the cæcum and the first inch or so of the colon. The tumor thus formed was about four inches in length and an inch and a half in diameter. The invaginated intestine was easily withdrawn through the ileo-cæcal valve, when it was found that about nine inches from the large gut the ileum was again invaginated into itself for a distance of two or three inches. It was impossible to completely reduce this, since the parts were much swollen and agglutinated by inflammatory lymph. The lumen of the gut was completely occluded just above and below this tumor. The intestine was in a fairly healthy condition. The invaginated portion, about five inches in length, was removed, and the ends of the intestine united by the Lembert suture. The mesentery, which had been cut off close to the bowel, was attached to the line of union of the intestine. The operation lasted an hour and a half, the child surviving it only a short time.

*Case II.*—Eight years ago my own son, a nursing baby of 12 months of age, while playing on the floor, a perfect picture of health and activity, suddenly cried out and began to strain as if at stool. The child had never been ill; his bowels had always acted regularly and naturally. This attack came on at 10 o'clock in the morning. It lasted but a moment, when he resumed his play. Similar attacks came on at intervals of about twenty minutes. When I saw him two hours later he had just had his first bloody passage. During the afternoon he voided several small passages stained with blood and mucus. About three o'clock Dr. G. W. Hutchins was called in, and under chloroform we administered a rectal injection of water, completely distending the colon throughout its entire length. The straining still continuing, Drs. Powell and Bogue were called in at 8 P. M., chloroform and rectal injection being repeated. The child rested quietly till 12 o'clock, midnight, when the straining began again. At 8 o'clock next morning Dr. Powell, assisted by Drs. Bogue, E. Ingals, Hutchins and the writer, opened the abdomen in the median line, and readily turned out the cæcum. About six or eight inches of the ileum had passed through the ileo-cæcal valve. The act of turning these parts out caused a backward peristaltic wave of the small intestines to take place and the invaginated portion was crowded out of the ileo-cæcal opening; it was not pulled out. The parts were only slightly congested. The abdomen was quickly closed and dressed. A natural passage of the bowels took place eight hours afterward. For ten days everything went well; the wound had healed. There was no peritonitis. He seemed to be convalescing. On the evening of the eleventh day slight twitchings of the muscles of the face were first observed. He died before morning in general convulsions.

In neither of the cases could any satisfactory cause of the invagination be given. Both children were remarkably healthy up to the very moment of the attack. In one there was a tendency to slight constipation. In the other, about two hours before the first symptom of trouble was present, the mother changed the diapers which had just been soiled by a perfectly free and normal motion. The child was always delighted to have his lower body free, and during this particular act of changing the tidy was possibly more hilarious than usual, kicking and crowing with the greatest glee. There was one peculiar motion he was accustomed to make on such occasions; it consisted in drawing his feet up by the sides of his head and forcibly throwing his legs downward across his mother's knee, thus bringing considerable sudden strain upon his lower abdomen. No other possible cause could be assigned, and this does not seem to be adequate to produce the trouble.

In operating on both these infants it was particularly noticeable how easily the cæcum was brought to the external surface through the median incision. It is well known that in the adult this cannot be easily done. Whether or not it is a normal condition for the cæcum of a child to be so much more movable than in the adult, I am unable to say. The text-books are silent on this point.

As to the treatment of these cases all agree that drugs are powerless. Surgery to be effective must be resorted to early. On the very first appearance of the symptoms indicating intestinal obstruction, rectal insufflation of hydrogen gas or atmospheric air should be resorted to at once. This plan is much better and safer than to fill the colon with water. The lightness and distensibility of these substances are in themselves enough to recommend them. Besides, water cannot be forced beyond the ileo-cæcal valve. On the other hand, Senn has demonstrated that the whole alimentary canal from anus to mouth can in nearly every case be easily and safely distended by rectal insufflation of hydrogen gas with a pressure varying from one-half to one and a half pounds. I am firmly convinced that had this plan been used in the second case here reported the intussusception would have been reduced without further operation.

If the apparatus for generating and using hydrogen gas is not at hand, air from the lungs may be blown into a rubber hot water bag or fountain syringe and so passed by a rectal tube into the anus. If, after a moderate trial of this method relief is not obtained, the knife must be resorted to at once.

Those of us who saw much of herniotomy for strangulation before the days of antiseptic surgery, have a very vivid recollection of the great mortality of the operation in comparison with its great success at the present time. The difference depends not so much upon modern technique as upon operating early before local changes have taken place in the constricted part. So in intestinal obstruction delay is dangerous. Do not wait for stercoracious vomiting, or to feel a lump in the iliac region. Open the abdomen in the median line, and do what you find there is to be done.

While I recommend in these cases rectal insufflation of hydrogen gas or air in preference to filling the gut with water, we must remember that not in all cases of perfectly pervious intestine will air distend the entire alimentary tract. I recently made laparotomy for gunshot wound of the abdominal cavity where before and after the incision we were unable to distend the bowel with air beyond the ileo-cæcal valve.

312 West Indiana Street.

